# Predictive biomarkers of resistance to hypofractionated radiotherapy in high grade glioma

**DOI:** 10.1186/s13014-017-0858-0

**Published:** 2017-07-28

**Authors:** Julian Biau, Emmanuel Chautard, Leanne De Koning, Frank Court, Bruno Pereira, Pierre Verrelle, Marie Dutreix

**Affiliations:** 1 Institut Curie, PSL Research University, Centre de Recherche, 91400 Orsay, France; 2 Institut Curie, PSL Research University, Centre de Recherche, 75248 Paris, France; 3 UMR3347, CNRS, 91400 Orsay, France; 4 U1021, INSERM, 91400 Orsay, France; 5 Université Paris Sud, 91400 Orsay, France; 6 Université Clermont Auvergne, INSERM, U1240 IMoST, F-63000 Clermont Ferrand, France; 7 Radiotherapy Department, Université Clermont Auvergne, Centre Jean Perrin, 58 rue Montalembert, 63011 Clermont-Ferrand, France; 8 Department of Translational Research, RPPA platform, Institut Curie, PSL Research University, 75248 Paris cedex05, France; 9 Université Clermont Auvergne, CNRS UMR 6293, INSERM U1103, GReD Laboratory, 63000 Clermont-Ferrand, France; 10 Biostatistics Department, DRCI, Clermont-Ferrand Hospital, 63003 Clermont-Ferrand, France; 11 U1196, INSERM, 91400 Orsay, France; 12 UMR9187, CNRS, 91400 Orsay, France

**Keywords:** High grade glioma, Radioresistance, RPPA, Hypofractionnated radiotherapy

## Abstract

**Background:**

Radiotherapy plays a major role in the management of high grade glioma. However, the radioresistance of glioma cells limits its efficiency and drives recurrence inside the irradiated tumor volume leading to poor outcome for patients. Stereotactic hypofractionated radiotherapy is one option for recurrent high grade gliomas. Optimization of hypofractionated radiotherapy with new radiosensitizing agents requires the identification of robust druggable targets involved in radioresistance.

**Methods:**

We generated 11 xenografted glioma models: 6 were derived from cell lines (1 WHO grade III and 5 grade IV) and 5 were patient derived xenografts (2 WHO grade III and 3 grade IV). Xenografts were treated by hypofractionated radiotherapy (6x5Gy). We searched for 89 biomarkers of radioresistance (39 total proteins, 26 phosphoproteins and 24 ratios of phosphoproteins on total proteins) using Reverse Phase Protein Array.

**Results:**

Both type of xenografted models showed equivalent spectrum of sensitivity and profile of response to hypofractionated radiotherapy. We report that Phospho-EGFR/EGFR, Phospho-Chk1/Chk1 and VCP were associated to resistance to hypofractionated radiotherapy.

**Conclusions:**

Several compounds targeting EGFR or CHK1 are already in clinical use and combining them with stereotactic hypofractionated radiotherapy for recurrent high grade gliomas might be of particular interest.

**Electronic supplementary material:**

The online version of this article (doi:10.1186/s13014-017-0858-0) contains supplementary material, which is available to authorized users.

## Background

Gliomas are the most frequent primary brain tumours in adults. According to WHO classification [1], molecular parameters and histology enable grading of the tumour as low grade (grade 2) or high grade (grade 3 or anaplastic gliomas; and grade 4 or glioblastomas) with increasing aggressiveness [2]. Major efforts have been made to identify relevant prognostic and predictive biomarkers in high grade gliomas including *IDH* (isocitrate dehydrogenase) mutations or ATRX (Alpha Thalassemia/mental Retardation syndrome X-linked) expression [3, 4]. Most alterations found in high grade gliomas target signalling pathways involved in invasion, signal transduction, cell-cycle control, DNA repair, angiogenesis or cell metabolism [5, 6].

The management of high grade gliomas includes maximal possible surgery because it reduces the symptoms from mass effect and probably improves survival. First line adjuvant therapy is based on systematic normofractionated radiotherapy (RT) (total dose of 60Gy; 5 fractions of 2Gy/week over 6 weeks), with daily temozolomide-based chemotherapy [7, 8]. Despite these treatments, median survival remains very low around 14 months. Early tumor relapses occur mostly within the high dose irradiated volume due to a high resistance of high grade glioma cells to RT [9]. Treatment options for recurrent high grade glioma are limited and hypofractionated stereotactic RT (HFRT) is one option with limited efficacy [10–14]. HFRT using fraction doses of 5–8 Gy provides normal tissue protection by its steep dose gradient while still providing the radiobiological potential advantage of high fraction doses. The reduced overall treatment time is an advantage for patients with short life expectancy. Therefore, a better understanding of biological mechanisms involved in high grade gliomas radioresistance to HFRT is a major issue for future drug development in the field of radiosensitization.

Common preclinical models for exploring cancer resistance to RT and radiosensitizing drugs include cultured human tumor cell lines in monolayer, xenografts derived from the same human cell lines or derived from patient samples grown subcutaneously in immunodeficient mice and orthotopic models [15, 16]. Although cell line xenograft models present only murine stroma, and minimal intra-tumoral heterogeneity, they constitute valuable in vivo models with high rates of successful engraftment, allowing a three-dimensional tumor cell proliferation.

High-throughput screening approaches are largely used to characterize cell lines and tumors and identify predictive biomarkers of treatment response [17]. The most currently used analyzed the RNA content (transcriptome) or DNA modification (genome) through dedicated microarrays or more recently high throughput sequencing. However in mammalian cells, a lot of regulatory modifications occur at post-transcriptional and translational levels and it is widely accepted that proteins amount and their modifications regulate cell signaling pathways that govern cell behavior, and their capacity to proliferate and escape from treatment [18, 19]. These differences could account for discrepancies sometimes reported between response to treatment in cell lines and results in vivo [16, 20]. However, despite the increasing interest for proteome, the methods to characterize it are still limited. In this study, we used reverse-phase protein array (RPPA), a technology using high-throughput antibody-based detection. It requires only a few μg of protein lysate and allows assessing protein expression and their main modification status in a highly quantitative manner [19, 21]. This technology can analyze hundreds of samples simultaneously on the same array and thus generate large datasets to identify potential diagnostic, prognostic, and predictive markers in human cancer [22].

The aim of this study was to search for predictive biomarkers of radioresponse on nude mice engrafted with high grade glioma cell lines and patient-derived tumor samples irradiated with a HFRT regimen. We explored a selection of proteins and modifications related to 10 different signaling pathways potentially involved in radioresistance: DNA repair [23], PI3K pathway [24], apoptosis [25], tyrosine kinase signaling [26], stress signaling, cell cycle [27], MAPK/ERK signaling, SAPK/JNK signaling [28], NFκB signaling [29] and adhesion/cytoskeleton [30].

## Methods

### Cell culture

SF763, SF767, and U87MG human glioma cell lines (Table 1) were given by Dr. C. Delmas (Centre Claudius Regaud, Toulouse, France). T98G, U118MG and CB193 cell lines (Table 1) were kindly provided by G. Pennarun (CEA, Grenoble, France). All culture reagents were purchased from GIBCO (Invitrogen, Cergy-Pontoise, France). Cells were grown in DMEM (with 4500 mg/l glucose and L-glutamine) supplemented with Sodium Pyruvate 1%, Non-Essential Amino Acids 1%, Gentamicin 10 μg/ml and 10% Fetal Calf Serum in a humidified incubator containing 5% CO2 at 37 °C.

**Table 1:** Radiosensitivity of in vivo glioma models

Name	Origin	Xenograft origin	Treatment	Number of mice	Median survival in days	Survival enhancement after RT (%)	*p*-value^a^	Mean TGD in days (%)	Quadrupling Time of RT vs NT *p*-value^b^
CB193	glioma grade III	Cell line	NT	9	42 [37;46]	226	< 0.001	66 ± 13 (351)	< 0.01
			RT	6	95 [88;140]				
SF763	glioblastoma	Cell line	NT	12	39 [32;52]	226	< 0.01	54 ± 15 (399)	< 0.001
			RT	7	88 [81;102]				
SF767	glioblastoma	Cell line	NT	8	77 [65;80]	168	< 0.001	52 ± 7 (215)	< 0.001
			RT	12	129 [101;140]				
T98G	glioblastoma	Cell line	NT	8	20 [16;23]	175	< 0.001	15 ± 5 (259)	< 0.01
			RT	8	35 [33;37]				
U87MG	glioblastoma	Cell line	NT	6	29 [22;30]	134	< 0.001	11 ± 1 (149)	< 0.01
			RT	10	39 [37;41]				
U118MG	glioblastoma	Cell line	NT	6	38 [23;49]	179	0.059	26 ± 4 (278)	< 0.01
			RT	9	68 [51;79]				
GBM-1-HAM	glioblastoma	Patient sample	NT	13	16 [14;18]	319	< 0.001	19 ± 4 (320)	< 0.001
			RT	9	51 [37;72]				
GBM-14-CHA	glioblastoma	Patient sample	NT	6	16 [14;19]	250	< 0.001	2 ± 1 (115)	ns
			RT	10	40 [28;44]				
GBM-14-RAV	glioblastoma	Patient sample	NT	6	29 [24;38]	259	< 0.001	37 ± 5 (386)	< 0.01
			RT	10	75 [61;82]				
ODA-17-GIR	glioma grade III	Patient sample	NT	6	19 [14;25]	405	< 0.001	55 ± 3 (715)	< 0.01
			RT	8	77 [65;79]				
ODA-4-GEN	glioma grade III	Patient sample	NT	6	26 [20;28]	215	< 0.001	33 ± 3 (462)	< 0.01
			RT	8	56 [51;63]				

Medians were presented with inter-quartile range [0.25; 0.75] and means with standard-errors

*ns* not significant, *NT* Not treated, *RT* Radiotherapy, *TGD* Tumor Growth Delay

^a^Log-rank test

^b^Mann Whitney Test

### Xenograft models

Xenografts derived from cell lines (CDX) were obtained by injecting 4 × 10^6^ cells of each cell line described above into the flank of adult female nude mice (Swiss nu/nu, Janvier, Le Genest Saint Isle, France). For patient derived xenograft (PDX), tumors are kept by successive grafting into scapular area or storage at −80 °C in fetal calf serum-free DMEM (Sigma-Aldrich, St Louis, MO, USA) for longer period. For each experiment, small fragments (50mm^3^) from scapular tumour were xenografted subcutaneously into the flank of anaesthetized nude mice (Swiss nu/nu, Janvier, Le Genest Saint Isle, France) aged 6–8 weeks. Xenografting was performed within 2 h following surgical resection, with fragments being kept in fetal calf serum-free DMEM (Sigma-Aldrich, St Louis, MO, USA) [31].

The animals were housed in our animal facility for at least 1 week before starting the experiments. There were six animals per cage under controlled conditions of light and dark cycles (12 h: 12 h), relative humidity (55%), and temperature (21 °C). Food and tap water were available ad libitum. When subcutaneous tumors reached approximately 125 mm^3^, mice were separated into homogeneous groups (at least 6 mice per group) to receive different treatment protocols: no treatment (NT) or HFRT alone for 2 weeks (HFRT: 6 × 5 Gy each Monday, Wednesday, Friday). A ^137^Cs unit (0.5 Gy/min) with a shield designed to protect approximately two thirds of the animal’s body was used to administer HFRT. Doses were controlled by thermoluminescence dosimetry. In all experiments, tumors were measured with a digital caliper every 2 to 3 days and any local skin toxicity was noted. Tumor volumes were calculated using the following formula: length × width × width/2. Mice were weighed every week and followed up for a maximum of 180 days. For ethical reasons, the animals were sacrificed when tumors reached 2000 mm3. The Local Committee on Ethics of Animal Experimentation approved all experiments.

### Antibodies and validation

The 65 antibodies used are listed in Additional file 1: Table S1. We explored 39 total proteins, 26 phosphoproteins and then calculated 24 ratios of phosphoproteins on total proteins. These proteins and modifications were chosen as representative of 10 different signaling pathways potentially involved in radioresistance: DNA repair, PI3K pathway, apoptosis, tyrosine kinase signaling, stress signaling, cell cycle, MAPK/ERK signaling, SAPK/JNK signaling, NFκB signaling and adhesion/cytoskeleton (Additional file 1: Table S1). To ensure that our antibodies were of sufficient quality, we confirmed their specificity by Western blotting on a large panel of cell lines treated with ligands and inhibitors. Antibodies with only a single or dominant band on Western blotting were validated and used in RPPA.

### Western blot

To ensure that our antibodies were of sufficient quality, we confirmed their specificity by Western blotting. For this, we used 14 cell lines from different origins, cultured in presence or absence of serum (inducing proliferation) and treated or not with bleocin (inducing DNA damage) or staurosporin (inducing apoptosis). 10 μg of lysate were run on precasted 4–15% polyacrylamide TGX gels (Bio-Rad). Transfer onto nitrocellulose was achieved using the Transblot Turbo (Bio-Rad). Membranes were blocked using TBS + 0.1% Tween +5% BSA. Primary antibodies were diluted in blocking solution and incubated overnight. Membranes were washed in TBS + 0.1% Tween and incubated for 1 h in secondary antibodies (Jackson ImmunoResearch), diluted in blocking solution. Membranes were washed and incubated with ECL (SuperSignal West Pico, Thermo Scientific) and signal was detected using a LAS3000 camera (Fuji). Antibodies with only a single or dominant band on Western blotting were validated and used in RPPA.

### Preparation of protein lysates from tumor tissue

Tumors were mechanically disrupted in 1 mL (for 80 mg of sample) of Laemmli Buffer (50 mM Tris pH = 6.8, 2% SDS, 5% glycerol, 2 mM DTT, 2,5 mM EDTA, 2,5 mM EGTA, 2× Perbio Halt Phosphatase inhibitor cocktail, Roche Protease inhibitors complete MINI EDTA-free, 4 mM Sodium Orthovanadate, 20 mM Sodium Fluoride) using a Precellys (Bertin Technologies, Montigny le Bretonneux, France) with CK28 beads at 4 °C, 6000 rpm, 60s three times. Extracts were boiled for 10 min at +100 °C and spinned 15 min at 13000 rpm at 15 °C. The protein fraction was transfered to a new tube and spinned again at 13000 rpm, for 5 min, at 15 °C. The pellet was discarded and lysated were snapfrozen. Protein concentration was measured using the Reducing Agent Compatible BCA kit (Pierce, Rockford, IL, USA).

### Reverse phase protein Array

Proteins were analyzed with 6 replicates for subcutaneous xenograft models (2 different locations in three different tumors per model). Samples were printed onto nitrocellulose-covered slides (Schott Nexterion NC-C, Jena, Germany) with a dedicated arrayer (2470 Arrayer, Aushon Biosystems, Billerica, MA, USA). Five serial dilutions, ranging from 2 to 0.125 mg/ml, and four technical replicates per dilution were used for each sample. Detection was carried out with specific antibodies or without primary antibody (negative control), using an Autostainer Plus (Dako, Trappes, France). Briefly, slides were incubated with avidin, biotin, and peroxidase blocking reagents (Dako, Trappes, France) and then saturated with TBS supplemented with 0.1% Tween 20 and 5% BSA (TBST-BSA). The slides were then probed by overnight incubation at 4 °C with primary antibodies diluted in TBST-BSA. The arrays were washed in TBST and then probed with horseradish peroxidase-coupled secondary antibodies (Jackson ImmunoResearch, Newmarket, UK) diluted in TBST-BSA for 1 h at room temperature. The signal was amplified by incubating the slides with amplification reagent (Bio-Rad, Cressier, Switzerland) for 15 min at room temperature. The arrays were then washed with TBST, probed with Cy5-streptavidin (Jackson ImmunoResearch, Newmarket, UK) diluted in TBST-BSA for 1 h at room temperature, and washed again in TBST. Total protein staining was achieved by incubating the arrays for 15 min in 7% acetic acid and 10% methanol, rinsing twice in water, incubating for 10 min in Sypro Ruby (Invitrogen, Cergy-Pontoise, France), and rinsing again. The processed slides were dried by centrifugation and scanned with a GenePix 4000B microarray scanner (Molecular Devices, Sunnyvale, CA, USA). Spot intensity was determined with MicroVigene software (VigeneTech Inc., Carlisle, MA, USA). Quantification of the data was achieved with NormaCurve [32], which includes data normalization against negative control slides and Sypro Ruby slides.

### Statistical analysis

Statistical analysis was performed using R v2.15.1 (http://www.cran.r-project.org). The tests were two-sided, with a Type I error set at α = 0.05. Continuous data were expressed according to sample size and to statistical distribution. So, medians were presented with inter-quartile range [0.25; 0.75] and means with standard-error. The study of relation between quantitative parameters was explored by Pearson or Spearman correlation coefficients r, according to statistical distribution (assumption of normality studied using Shapiro-Wilk test). To explore variations between PDX and CDX tumor growth before and after radiation, Mann-Whitney test were performed according to sample size and if assumptions of parametric test are not met (normality and homoscedasticity). Tumor growth delay (TGD) was calculated by subtracting the mean tumor volume quadrupling time of the control group from tumor volume quadrupling times of individual mice in each treatment group. The mean TGD was calculated for each treatment group from the individual measurements and was used to determine xenografts radioresponse. Survival curves were plotted according to the Kaplan–Meier method, and survival fraction of control and irradiated groups was compared using log-rank test analyses. *P*-value of less than or equal to 0.05 was considered to be a significant difference. Heatmaps were drawn using Gplots library.

## Results

### In vivo radiosensitivity determination

**Fig. 1:**
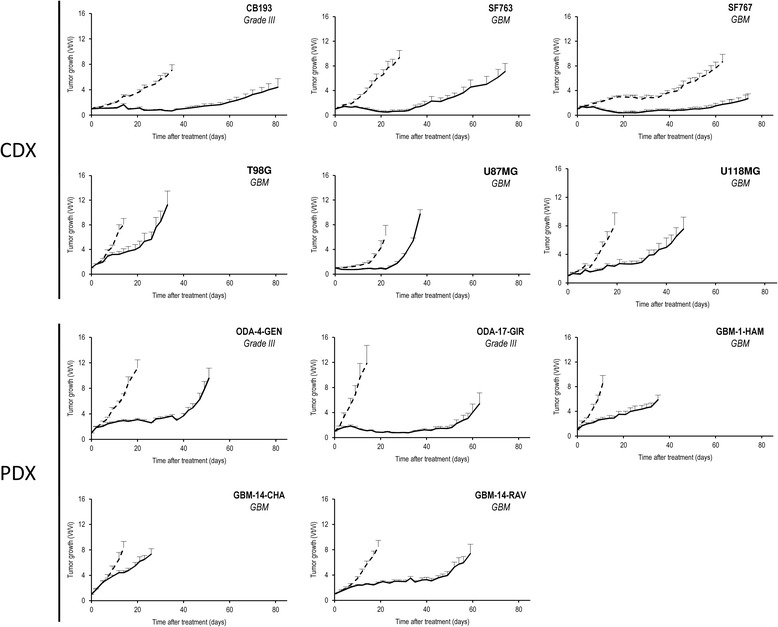
In vivo glioma models tumor growth after radiotherapy. Xenografts derived from cell lines (CDX) were obtained by injecting grade III or glioblastoma (GBM) cells into the flank of nude mice. For patient derived xenograft (PDX), each tumour was xenografted subcutaneously into the scapular area after a maximal delay of 2 h after surgical resection. Representation of tumour growth kinetics in NT group (dotted-line, *n* ≥ 6) and in group treated with 6 × 5 Gy (solid line, *n* ≥ 6)

CDX or PDX were generated to assess in vivo response to HFRT. Without irradiation, the 5 PDX had significantly faster tumor growth than the 6 CDX (mean tumor quadrupling time of 10 days vs 23 days, *p* < 0.05) and lower survival (median survival of 22 days vs 43 days, *p* < 0.05, Table 1). However, PDX and CDX had similar response after irradiation as the difference of median survival (around 20 days) was conserved after irradiation (59 days vs 78 days, *p* = 0.41, See Additional file 2: Fig. S1). TGD measures the difference between the mean time required to attain a four-times increase of irradiated tumor volume as compared to non-irradiated controls (Fig. 1). TGD should not be highly sensitive to tumor growth speed. In most tumor models, HFRT temporarily blocks tumor growth, with a tumor growth arrest after the end treatment (10 days) that remains stable for up to 40 days (the best responder being ODA-17-GIR, GBM-14-RAV, CB193, SF767). The GBM-14-CHA did not show any growth arrest after HFRT. HFRT enhanced survival of all engrafted mice (fold increase of median survival ranged from 140 to 390%, Table 1). Mean TGD and median survival were correlated (*p* = 0.005, *r* = 0.78). GBM-14-CHA had the lowest TGD and thus was considered as the most radioresistant xenograft while ODA-17-GIR had the highest TGD and thus was the most radiosensitive (Table 1).

### Protein biomarkers of radioresistance

We used an RPPA approach to identify biomarkers of radioresponse in glioma subcutaneous xenografts (*n* = 11). A total of 89 protein markers: 39 total proteins, 26 phosphoproteins and 24 calculated ratios of phosphoproteins on total proteins were analyzed (See Additional file 1: Table S1). To take into account intrinsic variations, 6 different tumor samples were analyzed per model. We did not find significant variations between samples outlining little heterogeneity of biological replicates [33].

Firstly, we compared protein profile of CDX and PDX using unsupervised hierarchical clustering of the RPPA data (See Additional file 3: Fig. S2). There was no clear difference between protein profiles of tumors issued from CDX or PDX grafting. Protein patterns were not dependent on the type of graft (CDX vs PDX), but rather vary between models. Indeed, no protein was found significantly differentially expressed between CDX and PDX models.

**Fig. 2:**
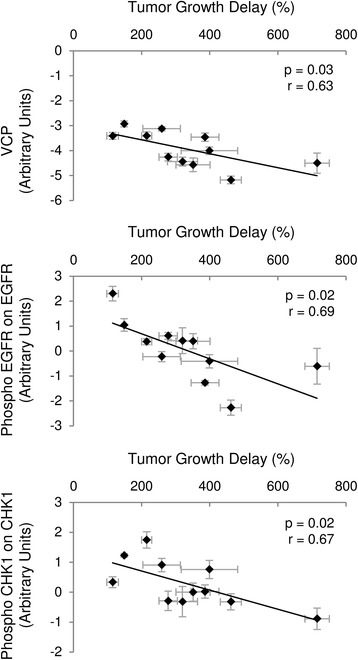
Markers associated with response to irradiation. Identified biomarkers for in vivo response to irradiation. Protein level (arbitrary units) is plot together with Tumor Growth Delay(%). Data are presented by their mean ± standard error

Then, we looked for proteins biomarkers of radioresponse in vivo. For this, we sought to explore the relationship between TGD and the 89 protein markers previously obtained by RPPA assay. Interestingly, the markers specific of in vivo resistance to radiotherapy were Phospho-EGFR (Tyr1173) on EGFR (*p* = 0.02, *r* = 0.69), Phospho-Chk1 (Ser280) on Chk1 (*p* = 0.02, *r* = 0.67) and the VCP (valosine-containing protein, *p* = 0.03, *r* = 0.63), which is a chaperone protein that regulates ubiquitin-dependent protein degradation. In vivo biomarkers were positively correlated to radioresistance with a high level being associated to more resistance (Fig. 2).

## Discussion

Despite encouraging preclinical results, and numerous clinical trials [34–37], only one targeted therapy has so far been approved for clinical use in combination with RT in head and neck cancer [38] and none in high grade glioma treatment. Therefore, a better understanding of the mechanisms involved in RT resistance of a type of cancer (theranostic biomarkers) is necessary prior to proposing combined treatments with RT. Optimization of RT efficiency is one of the most promising approaches to increase patient survival notably high grade glioma. Stereotactic HFRT is appealing in the treatment of recurrent high grade glioma because of its ability to accurately deliver high doses of RT in a single or several fractions and to spare surrounding normal brain tissue [10–14]. Median survival in these series was between 7 and 13 months from time of salvage treatment with acceptable toxicity. However, despite recent developments in the spatial targeting of HFRT [39, 40], the inability to obtain long term control of diseases emphasizes the need to overcome tumor radioresistance with new agents that target molecular and cellular pathways underlying the mechanisms of such resistance. Here we aimed to look for predictive in vivo biomarkers of glioma radioresistance to HFRT in order to identify potential druggable targets. We used sub-cutaneous xenografts, rather than orthotopic in vivo model because subcutaneous xenograft models are very convenient to avoid radiation side effects and to monitor accurately tumor growth [41, 42]. Furthermore, we studied the response to a HFRT protocol currently used in high grade glioma reirradiation [10, 13] (6 fractions of 5 Gy over 2 weeks) that could not be ethically studied in orthopic xenografts due to its potential high level of toxicity in mice [43]. One bias of our analysis might be that HFRT has been applied in previously unirradiated xenografts. Initial irradiation might affect tumor bed microenvironment such as blood vessels, and effects of re-irradiation with HFRT might be influenced by that. However, it is impossible and unethical to reproduce the conditions used in clinics in mice models both concerning initial total dose of radiation therapy (60Gy in 30 fractions) and the delay between initial irradiation and re-irradiation (>6–12 months).

As RT cytotoxicity is mainly due to DNA damage, we explored the correlation between DNA repair enzymes status and survival to irradiation. Several studies suggested that overexpression/activation of H2AX, 53BP1 or P53 could be associated to radioresistance [44, 45] but none of this studies addressed the specific question of high grade glioma radioresistance to HFRT. Expression of most of proteins directly involved in DNA repair were not significantly different between sensitive and resistant xenografts. We did not find any correlation between the level of DNA-PK, a key player in NHEJ double-strand break repair, and radiosensitivity in xenografts. However, VCP, a potentially regulator of DNA-PK activity, was associated with in vivo radioresponse to HFRT. VCP is a chaperone protein that regulates ubiquitin-dependent protein degradation [46]. VCP could play a dual role in regulation of DNA repair by controlling the proteasome-mediated degradation of DNA-PK [47] and acting at the damage site where it is phosphorylated by DNA-PK [48] and interacts with several DNA repair and cell cycle proteins [49–51].

Two other proteins, the epidermal growth factor receptor (EGFR) and the checkpoint kinase (CHK1) were associated with in vivo radioresistance to HFRT, and could thus be potential targets for radiosensitization. CHK1 plays an important role in the DNA damage response to radiation (including cell cycle control) and has been notably involved in the resistance of glioma stem like cells to ionizing radiation [27, 52]. The interest for CHK1 targeting in cancer area is underlined by the use of several compounds (CCT245737, LY2606368, Prexasertib) in ongoing clinical trials, though none of them explore the association with radiotherapy.

EGFR, which was highly correlated to tumor response in our study has been found overexpressed in about 40% of glioblastomas [53] with approximately 50% of tumors with *EGFR* amplification carrying a specific EGFR mutant (EGFRvIII) [26]. EGFR signaling (belonging to tyrosine kinase signaling) is involved in growth signaling, differentiation, adhesion, migration and survival of cancer cells [54]. More recent studies highlighted a direct link between EGFR signaling and DNA repair. Thus targeting EGFR signaling could be a promising way to radiosensitize human glioma [55, 56]. Prados et al. reported outcomes of a phase 2 study of EGFR inhibitor erlotinib + Temozolomide during and after RT in patients with newly diagnosed glioblastoma [34]. Median survival was 19.3 months vs 14.1 months in the historical cohorts. However, in a phase 1/2 study of EGFR inhibitor gefitinib during and after RT, Chakravarti et al. did not find any survival benefit with historical cohort [57]. Further clinical investigations are needed. A phase 2 trial is actually ongoing (NCT02800486) and addresses the specific question of the combination of EGFR inhibitor cetuximab with stereotactic HFRT for the treatment of previously irradiated recurrent high grade glioma.

## Conclusions

Our study is one of the first studies to address the specific question of protein biomarkers of resistance to HFRT in high grade gliomas. We found 3 biomarkers of particular interest: VCP, EGFR and CHK1. Several compounds targeting EGFR or CHK1 are already in clinical use and our study suggest that combining these compounds with stereotactic HFRT for recurrent high grade gliomas might be of particular interest.

## Additional files

Additional file 1: Table S1. Antibodies used in RPPA assay. (XLSX 18 kb)

Additional file 2: Fig. S1. In vivo glioma models survival after radiotherapy. Xenografts derived from cell lines (CDX) were obtained by injecting grade III or glioblastoma (GBM) cells into the flank of nude mice. For patient derived xenograft (PDX), each tumour was xenografted subcutaneously into the scapular area after a maximal delay of 2 h after surgical resection. Survival curves of NT group (dotted-line, *n* ≥ 6) and group treated with 6 × 5 Gy (solid line, *n* ≥ 6) were plotted according to the Kaplan–Meier method, and survival fraction of groups was compared using log-rank test. (TIFF 1691 kb)

Additional file 3: Fig. S2. Heatmap of the RPPA data for CDX and PDX. Data obtained for 5 PDX (Green) and 6 CDX (Blue) were used (6 relicates) by unsupervised hierarchical clustering using Gplots library in R software. Expression for the 65 proteins or phosphor-proteins studied range from low (white) to high (black). (TIFF 2112 kb)

## Abbreviations

ATRX: Alpha Thalassemia/mental Retardation syndrome X-linked
CDX: Xenografts derived from cell lines
CHK1: Checkpoint 1
EGFR: Epidermal growth factor receptor
HFRT: Hypofractionated radiotherapy
IDH: Isocitrate dehydrogenase
NHEJ: Non-homologous-end-joining
NT: No treatment
PDX: Patient derived xenograft
RPPA: Reverse-phase protein array
RT: Radiotherapy
TGD: Tumor growth delay
VCP: Valosine-containing protein

## References

[1] Louis DN (2006). Molecular pathology of malignant gliomas. Annu Rev Pathol Mech Dis.

[2] Ricard D, Idbaih A, Ducray F, Lahutte M, Hoang-Xuan K, Delattre J-Y (2012). Primary brain tumours in adults. Lancet Lond Engl.

[3] Cohen AL, Holmen SL, Colman H (2013). IDH1 and IDH2 mutations in Gliomas. Curr Neurol Neurosci Rep.

[4] Walsh KM, Wiencke JK, Lachance DH, Wiemels JL, Molinaro AM, Eckel-Passow JE (2015). Telomere maintenance and the etiology of adult glioma. Neuro-Oncol.

[5] Cancer Genome Atlas Research Network (2008). Comprehensive genomic characterization defines human glioblastoma genes and core pathways. Nature.

[6] Wang H, Xu T, Jiang Y, Xu H, Yan Y, Fu D (2015). The challenges and the promise of molecular targeted therapy in malignant gliomas. Neoplasia N Y N.

[7] Stupp R, Hegi ME, Mason WP, van den Bent MJ, Taphoorn MJB, Janzer RC (2009). Effects of radiotherapy with concomitant and adjuvant temozolomide versus radiotherapy alone on survival in glioblastoma in a randomised phase III study: 5-year analysis of the EORTC-NCIC trial. Lancet Oncol.

[8] Stupp R, Mason WP, van den Bent MJ, Weller M, Fisher B, Taphoorn MJB (2005). Radiotherapy plus concomitant and adjuvant temozolomide for glioblastoma. N Engl J Med.

[9] Bleehen NM, Stenning SP (1991). A Medical Research Council trial of two radiotherapy doses in the treatment of grades 3 and 4 astrocytoma. The Medical Research Council brain tumour working party. Br J Cancer.

[10] Grosu AL, Weber WA, Franz M, Stärk S, Piert M, Thamm R (2005). Reirradiation of recurrent high-grade gliomas using amino acid PET (SPECT)/CT/MRI image fusion to determine gross tumor volume for stereotactic fractionated radiotherapy. Int J Radiat Oncol Biol Phys.

[11] Khalil T, Lemaire J-J, Dedieu V, Donnarieix D (2013). MRI tumor response and clinical outcomes after LINAC radiosurgery on 50 patients with recurrent malignant gliomas. J Radiosurgery SBRT.

[12] McKenzie JT, Guarnaschelli JN, Vagal AS, Warnick RE, Breneman JC (2013). Hypofractionated stereotactic radiotherapy for unifocal and multifocal recurrence of malignant gliomas. J Neuro-Oncol.

[13] Vordermark D, Kölbl O, Ruprecht K, Vince GH, Bratengeier K, Flentje M (2005). Hypofractionated stereotactic re-irradiation: treatment option in recurrent malignant glioma. BMC Cancer.

[14] Yazici G, Cengiz M, Ozyigit G, Eren G, Yildiz F, Akyol F (2014). Hypofractionated stereotactic reirradiation for recurrent glioblastoma. J Neuro-Oncol.

[15] Stylli SS, Luwor RB, Ware TMB, Tan F, Kaye AH (2015). Mouse models of glioma. J Clin Neurosci.

[16] Marx V (2014). Models: stretching the skills of cell lines and mice. Nat Methods.

[17] Hayes DF (2015). Biomarker validation and testing. Mol Oncol.

[18] Henry NL, Hayes DF (2012). Cancer biomarkers. Mol Oncol.

[19] Gallagher RI, Espina V (2014). Reverse phase protein arrays: mapping the path towards personalized medicine. Mol Diagn Ther.

[20] Hughes J, Rees S, Kalindjian S, Philpott K (2011). Principles of early drug discovery. Br J Pharmacol.

[21] Masuda M, Yamada T (1854). Signaling pathway profiling by reverse-phase protein array for personalized cancer medicine. Biochim Biophys Acta.

[22] Akbani R, Becker K-F, Carragher N, Goldstein T, de Koning L, Korf U (2014). Realizing the promise of reverse phase protein arrays for clinical, translational, and basic research: a workshop report: the RPPA (reverse phase protein array) society. Mol Cell Proteomics MCP.

[23] Helleday T, Petermann E, Lundin C, Hodgson B, Sharma RA (2008). DNA repair pathways as targets for cancer therapy. Nat Rev Cancer.

[24] Chautard E, Loubeau G, Tchirkov A, Chassagne J, Vermot-Desroches C, Morel L (2010). Akt signaling pathway: a target for radiosensitizing human malignant glioma. Neuro-Oncol.

[25] Maier P, Hartmann L, Wenz F, Herskind C. Cellular pathways in response to ionizing radiation and their targetability for tumor radiosensitization. Int J Mol Sci. 2016;17:102. 10.3390/ijms17010102 PMC4730344 26784176

[26] Nyati MK, Morgan MA, Feng FY, Lawrence TS (2006). Integration of EGFR inhibitors with radiochemotherapy. Nat Rev Cancer.

[27] Burdak-Rothkamm S, Rothkamm K, McClelland K, Al Rashid ST, Prise KM (2015). BRCA1, FANCD2 and Chk1 are potential molecular targets for the modulation of a radiation-induced DNA damage response in bystander cells. Cancer Lett.

[28] Tang FR, Loke WK (2015). Molecular mechanisms of low dose ionizing radiation-induced hormesis, adaptive responses, radioresistance, bystander effects, and genomic instability. Int J Radiat Biol.

[29] Bai M, Ma X, Li X, Wang X, Mei Q, Li X (2015). The accomplices of NF-κB lead to Radioresistance. Curr Protein Pept Sci.

[30] Satelli A, Li S (2011). Vimentin in cancer and its potential as a molecular target for cancer therapy. Cell Mol Life Sci CMLS.

[31] Leuraud P, Taillandier L, Aguirre-Cruz L, Medioni J, Crinière E, Marie Y (2003). Correlation between genetic alterations and growth of human malignant glioma xenografted in nude mice. Br J Cancer.

[32] Troncale S, Barbet A, Coulibaly L, Henry E, He B, Barillot E (2012). NormaCurve: a SuperCurve-based method that simultaneously quantifies and normalizes reverse phase protein array data. PLoS One.

[33] Biau J, Chautard E, Court F, Pereira B, Verrelle P, Devun F (2016). Global conservation of protein status between cell lines and Xenografts. Transl Oncol.

[34] Prados MD, Chang SM, Butowski N, DeBoer R, Parvataneni R, Carliner H (2009). Phase II study of erlotinib plus temozolomide during and after radiation therapy in patients with newly diagnosed glioblastoma multiforme or gliosarcoma. J Clin Oncol Off J Am Soc Clin Oncol.

[35] Chinot OL, Wick W, Mason W, Henriksson R, Saran F, Nishikawa R (2014). Bevacizumab plus radiotherapy-temozolomide for newly diagnosed glioblastoma. N Engl J Med.

[36] Gutin PH, Iwamoto FM, Beal K, Mohile NA, Karimi S, Hou BL (2009). Safety and efficacy of bevacizumab with hypofractionated stereotactic irradiation for recurrent malignant gliomas. Int J Radiat Oncol.

[37] Couñago F, Rodríguez A, Calvo P, Luna J, Monroy JL, Taboada B, et al. Targeted therapy combined with radiotherapy in non-small-cell lung cancer: a review of the oncologic group for the study of lung cancer (Spanish radiation oncology society). Clin Transl Oncol. 2017;19(1):31-43. 10.1007/s12094-016-1512-2 27106020

[38] Bonner JA, Harari PM, Giralt J, Azarnia N, Shin DM, Cohen RB (2006). Radiotherapy plus cetuximab for squamous-cell carcinoma of the head and neck. N Engl J Med.

[39] Kollar L, Rengan R (2014). Stereotactic body radiotherapy. Semin Oncol.

[40] Rubio C, Morera R, Hernando O, Leroy T, Lartigau SE (2013). Extracranial stereotactic body radiotherapy. Review of main SBRT features and indications in primary tumors. Rep Pract Oncol Radiother J Gt Cancer Cent Pozn Pol Soc Radiat Oncol.

[41] de Vries NA, Beijnen JH, van Tellingen O (2009). High-grade glioma mouse models and their applicability for preclinical testing. Cancer Treat Rev.

[42] Fomchenko EI, Holland EC (2006). Mouse models of brain tumors and their applications in preclinical trials. Clin Cancer Res Off J Am Assoc Cancer Res.

[43] Workman P, Aboagye EO, Balkwill F, Balmain A, Bruder G, Chaplin DJ (2010). Guidelines for the welfare and use of animals in cancer research. Br J Cancer.

[44] Markova E, Vasilyev S, Belyaev I (2015). 53BP1 foci as a marker of tumor cell radiosensitivity. Neoplasma.

[45] Menegakis A, von Neubeck C, Yaromina A, Thames H, Hering S, Hennenlotter J (2015). γH2AX assay in ex vivo irradiated tumour specimens: a novel method to determine tumour radiation sensitivity in patient-derived material. Radiother Oncol J Eur Soc Ther Radiol Oncol.

[46] Meyer H, Weihl CC (2014). The VCP/p97 system at a glance: connecting cellular function to disease pathogenesis. J Cell Sci.

[47] Jiang N, Shen Y, Fei X, Sheng K, Sun P, Qiu Y (2013). Valosin-containing protein regulates the proteasome-mediated degradation of DNA-PKcs in glioma cells. Cell Death Dis.

[48] Livingstone M, Ruan H, Weiner J, Clauser KR, Strack P, Jin S (2005). Valosin-containing protein phosphorylation at Ser784 in response to DNA damage. Cancer Res.

[49] Zhang H, Wang Q, Kajino K, Greene MI (2000). VCP, a weak ATPase involved in multiple cellular events, interacts physically with BRCA1 in the nucleus of living cells. DNA Cell Biol.

[50] Acs K, Luijsterburg MS, Ackermann L, Salomons FA, Hoppe T, Dantuma NP (2011). The AAA-ATPase VCP/p97 promotes 53BP1 recruitment by removing L3MBTL1 from DNA double-strand breaks. Nat Struct Mol Biol.

[51] Meerang M, Ritz D, Paliwal S, Garajova Z, Bosshard M, Mailand N (2011). The ubiquitin-selective segregase VCP/p97 orchestrates the response to DNA double-strand breaks. Nat Cell Biol.

[52] Bao S, Wu Q, McLendon RE, Hao Y, Shi Q, Hjelmeland AB (2006). Glioma stem cells promote radioresistance by preferential activation of the DNA damage response. Nature.

[53] Ohgaki H, Kleihues P (2007). Genetic pathways to primary and secondary glioblastoma. Am J Pathol.

[54] De Luca A, Carotenuto A, Rachiglio A, Gallo M, Maiello MR, Aldinucci D (2008). The role of the EGFR signaling in tumor microenvironment. J Cell Physiol.

[55] Palumbo S, Tini P, Toscano M, Allavena G, Angeletti F, Manai F (2014). Combined EGFR and autophagy modulation impairs cell migration and enhances radiosensitivity in human glioblastoma cells. J Cell Physiol.

[56] Thorne AH, Zanca C, Furnari F (2016). Epidermal growth factor receptor targeting and challenges in glioblastoma. Neuro-Oncol.

[57] Chakravarti A, Wang M, Robins HI, Lautenschlaeger T, Curran WJ, Brachman DG (2013). RTOG 0211: a phase 1/2 study of radiation therapy with concurrent gefitinib for newly diagnosed glioblastoma patients. Int J Radiat Oncol Biol Phys.

