# Supplementary figures and images for: Predictive biomarkers of resistance to hypofractionated radiotherapy in high grade glioma
Source: Radiat Oncol. 2017 Jul 28;12:123. doi: 10.1186/s13014-017-0858-0 (PMC5534104; doi:10.1186/s13014-017-0858-0)

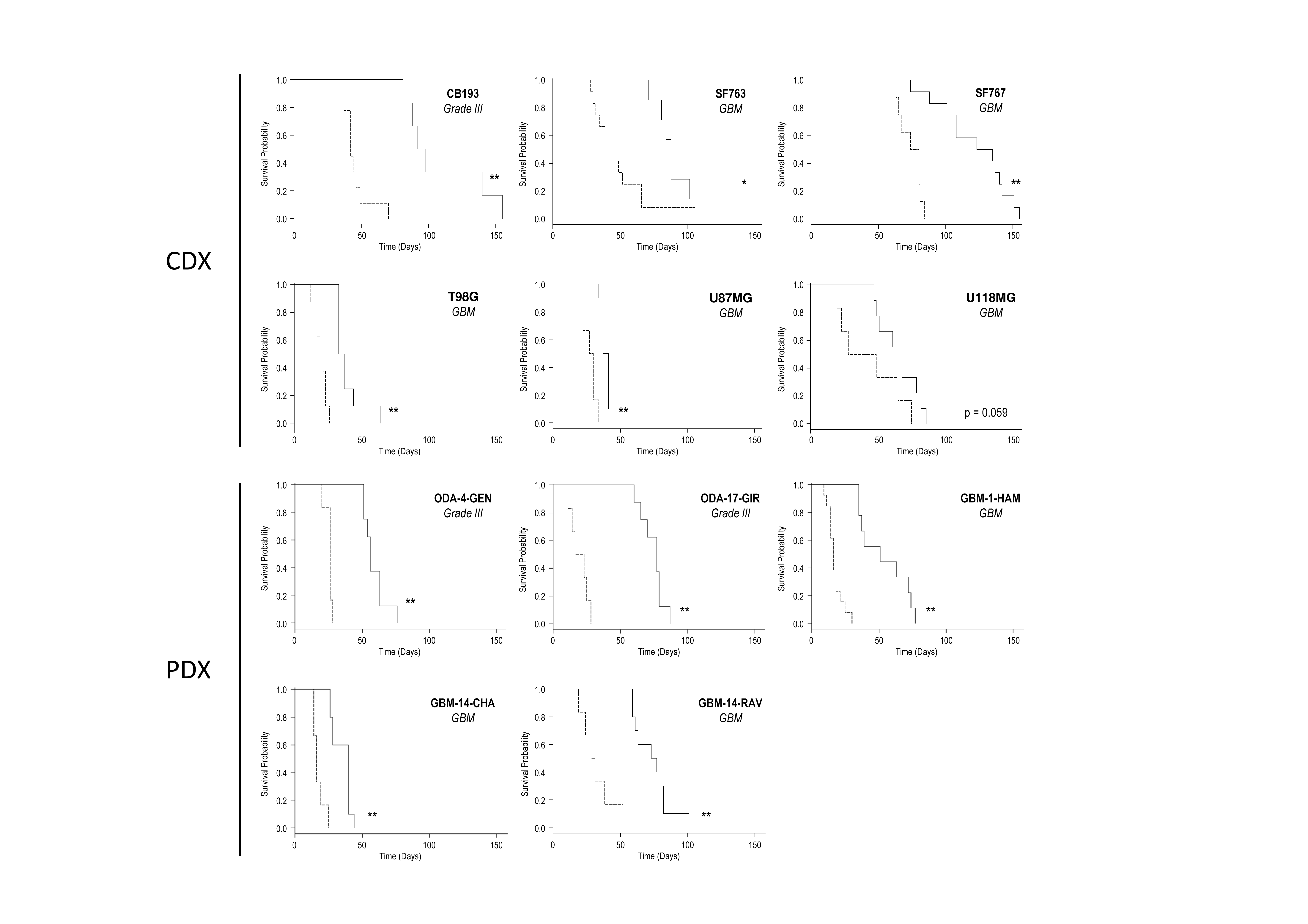

Supplement: Supplementary file 2 — In vivo glioma models survival after radiotherapy. Xenografts derived from cell lines (CDX) were obtained by injecting grade III or glioblastoma (GBM) cells into the flank of nude mice. For patient derived xenograft (PDX), each tumour was xenografted subcutaneously into the scapular area after a maximal delay of 2 h after surgical resection. Survival curves of NT group (dotted-line, n ≥ 6) and group treated with 6 × 5 Gy (solid line, n ≥ 6) were plotted according to the Kaplan–Meier method, and survival fraction of groups was compared using log-rank test. (TIFF 1691 kb) [file 13014_2017_858_MOESM2_ESM.tif]

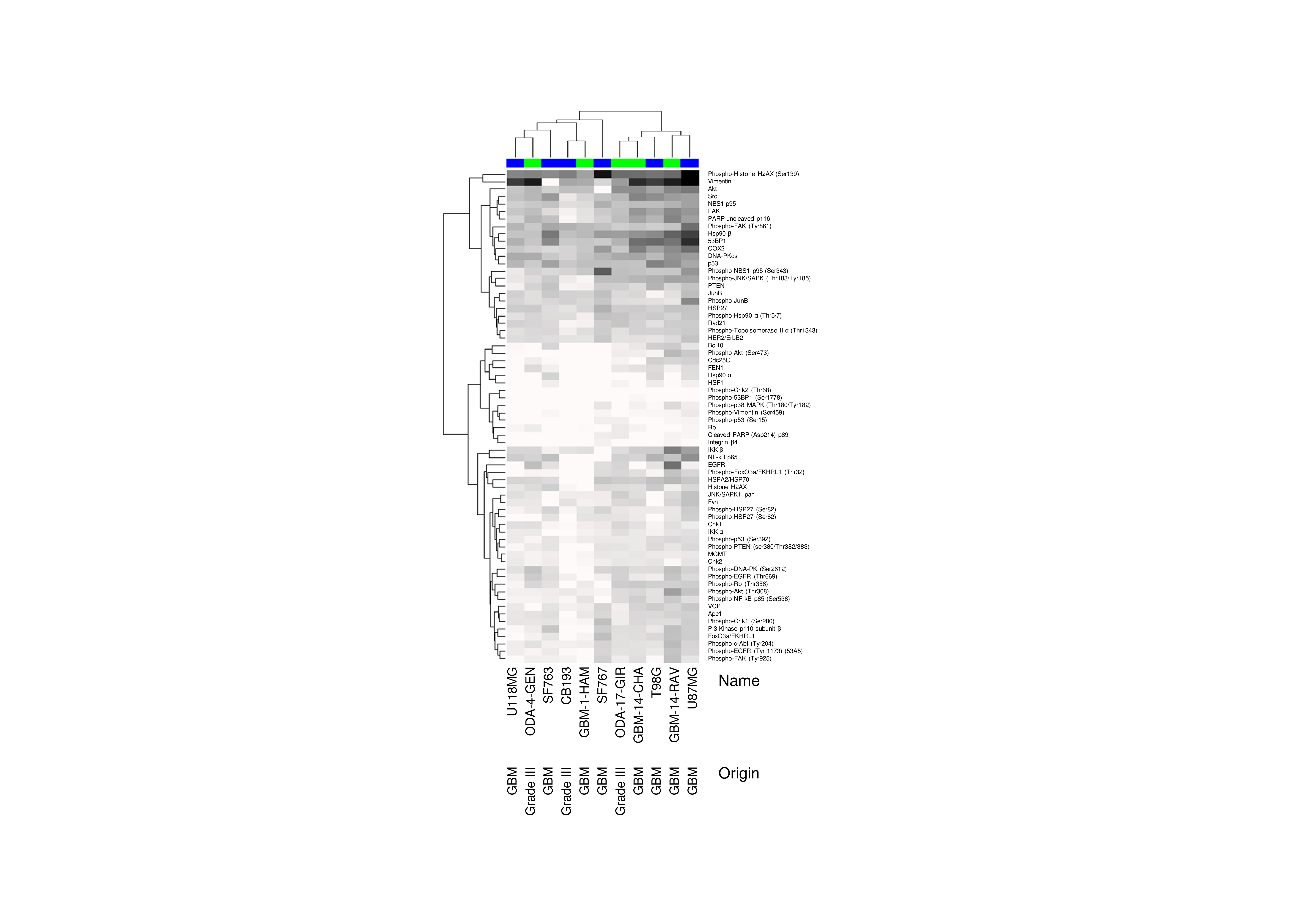

Supplement: Supplementary file 3 — Heatmap of the RPPA data for CDX and PDX. Data obtained for 5 PDX (Green) and 6 CDX (Blue) were used (6 relicates) by unsupervised hierarchical clustering using Gplots library in R software. Expression for the 65 proteins or phosphor-proteins studied range from low (white) to high (black). (TIFF 2112 kb) [file 13014_2017_858_MOESM3_ESM.tif]
